# Impact on Plankton Communities Following Abandonment of Rice Cultivation and Biotope Creation

**DOI:** 10.1002/ece3.72575

**Published:** 2025-12-21

**Authors:** Mariko Nagano, Ryoma Teramoto

**Affiliations:** ^1^ Kyoto University of Advanced Science (KUAS) Kyoto Japan

**Keywords:** biotope, land use, plankton community, rice paddy, Trinema

## Abstract

The global decline in agricultural land has raised concerns regarding the impact on biodiversity. One effective strategy for preventing a decrease in biodiversity is converting abandoned rice fields into wetland biotopes. However, the impact of these changes on plankton communities remains unclear. The objective of this study was to investigate the diversity and distribution of plankton communities in a single rice field located in Kyoto. The specific objective is to clarify which plankton respond to farmland abandonment. Samples were collected every week from May to July over a two‐year period (rice cultivation period in 2022 and post‐abandonment biotope creation in 2023) to determine species composition and environmental variation. As a result, no changes in plankton community structure occurred after biotope formation, as evidenced by the absence of statistically significant changes in the biodiversity index (*H′*) or the number of plankton taxa. At the genus level, *Trinema*, *Trachelomonas*, and *Scenedesmus* exhibited a response to fluctuations over the two‐year period, despite their low average contribution to community structure. These groups of organisms may be driving subtle changes in community structure and should therefore be considered as indicator species. Subsequent research endeavors will ascertain the true value of biotope formation in abandoned land.

## Graphical abstract

This study clarified changes in plankton communities during the first year of fallow in long‐term cultivated paddy fields in Kyoto. These fields were in a fallow state, with no planting, cultivation, or fertilization occurring. When water was introduced at the same time of year as during active use and the fields were converted into biotopes, diversity and species richness remained unchanged, but changes were observed in testate amoebae.

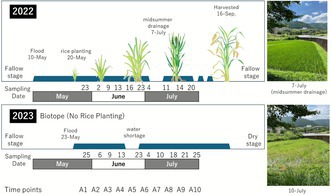

## 1 Introduction

In recent years, it has become increasingly difficult to analyze the factors associated with the decline in biodiversity. One of the main impacts on biodiversity is the loss of habitats due to changes in land use, the extent of which varies globally (Davison et al. 2021; Jaureguiberry et al. 2022). Land‐use change involving agricultural decline or abandonment can sometimes enhance biodiversity, particularly when natural regeneration persists (Outhwaite et al. 2022; Benayas et al. 2007). While farmland abandonment may offer opportunities for habitat regeneration, scientists view these as limited. Crucially, despite the need to assess biodiversity pre‐ and post‐abandonment, such studies remain scarce (Queiroz et al. 2014).

Biodiversity is frequently sustained not in spite of, but because of, traditional or sustainable human practices. For example, semi‐natural grasslands in northern Europe, maintained through traditional grazing and mowing, support exceptionally rich biota (Eriksson 2021; Storch et al. 2022). Similarly, in Mediterranean and Asian regions, long‐standing, low‐intensity agricultural practices have contributed to the maintenance of native biodiversity. The prominent example is the rice paddies, initially developed over 2000 years ago through the modification of natural wetlands. Today, rice paddies are shallow, seasonally flooded habitats that support a wide range of aquatic and semi‐aquatic organisms. In Asia, rice paddies are especially valued for supporting diverse communities of birds and endangered insects, and over 300 seasonally flooded habitats, including rice fields, have been registered under the Ramsar Convention (https://rsis.ramsar.org). In Japan, a database containing 6305 species of organisms found in rice paddies and their surrounding environments has been made publicly available (Database of Biota in Rice Paddies and Adjacent Areas 2020, https://www.biwahaku.jp/study/tambo/). The importance of rice fields in biodiversity is that traditional rice‐growing techniques and organisms have adapted over a long period of time. For example, there is a collapse of the communities in artificially created temporary shallow water, such as rice fields and irrigation canals. However, because this process is repeated over several years, it allows for the existence of organisms that require dryness, such as plankton, that have adapted to this process, such as by having resting eggs (Brown et al. 2022; O'Neill and Thorp 2014). Now, such communities are at risk due to agricultural intensification, land‐use conversion, and the increasing abandonment of traditional cultivation.

The abandonment of cultivation, rapid conversion to modern farming methods, and increase in these methods are endangering the biodiversity of agricultural land. To maintain biodiversity, some efforts are being made to develop abandoned rice fields as biotopes (Furukori and Homma 2024; Watanabe et al. 2024). These fallow rice paddy biotopes, functioning as constructed wetlands, are considered a conservation strategy that can mitigate abrupt land‐use changes and maintain biodiversity. To date, few studies have closely tracked the dynamics of biological communities during the transition of abandoned paddy fields into different ecosystems through artificial management (conversion into biotopes). One of the most interesting cases involved a comparison of mosquito larvae in fallow fields and adjacent paddy fields. While species richness and abundance were similar, the number of predatory mosquito species and their abundance tended to be higher in the biotope (Ohba et al. 2012). Even if fallow fields show no change in species number or density compared to adjacent paddy fields, the functional differences in mosquito larvae may still alter the plankton community at the lower trophic levels of the food web. Capturing subtle changes in community structure that are not apparent in diversity, species richness, or density—that is, changes without significant statistical differences—enables a more realistic understanding and assessment of ecosystem function. Several studies have examined plankton in rice paddies (phytoplankton, Fonge et al. 2012; phytoplankton and periphyton, Awasthi 2021; aquatic organisms, Kimura 2005; Yamazaki et al. 2003, 2004; zooplankton, Chittapun et al. 2009; resting egg of zooplankton, Plangklang and Athibai 2023), but none have clarified how these communities change when land use is converted to a biotope. This study aims to address this issue by comparing the biological community structures of converted paddy fields and identifying their qualitative changes.

Given the rapid pace of land‐use change, capturing comprehensive records of biodiversity before and after the cessation of agricultural activities is increasingly challenging. In Japan, rice field area is declining by 1% annually, equating to a loss of ~25,000 ha per year (MAFF 2023). A meta‐analysis found that the abandonment of rice fields reduces biodiversity, with an average decline in species richness and abundance of 56% (Koshida and Katayama 2018). Consequently, the practice of flooding fallow paddies to create artificial wetlands is gaining recognition as a biodiversity conservation strategy in Japan (Watanabe et al. 2024; Kato et al. 2025).

Our study elucidates the alternations in the plankton community changes during the initial year of fallowing in a long‐cultivated rice field in Kyoto. The field has been left fallow, with no planting, cultivation, fertilization, or pesticide application, and no tilling was performed. In Japan, the practice of flooding fallow paddies to create biotope is gaining attention as a strategy to mitigate abrupt environmental changes for the rice field community. This approach is believed to contribute to the preservation of biodiversity. This study aims to clarify whether the change in land use from paddy fields, which are considered to be highly diverse, to artificial wetlands is disruptive or restorative for the plankton community in the same area.

## 2 Materials and Methods

### 2.1 Study Site

**FIGURE 1:**
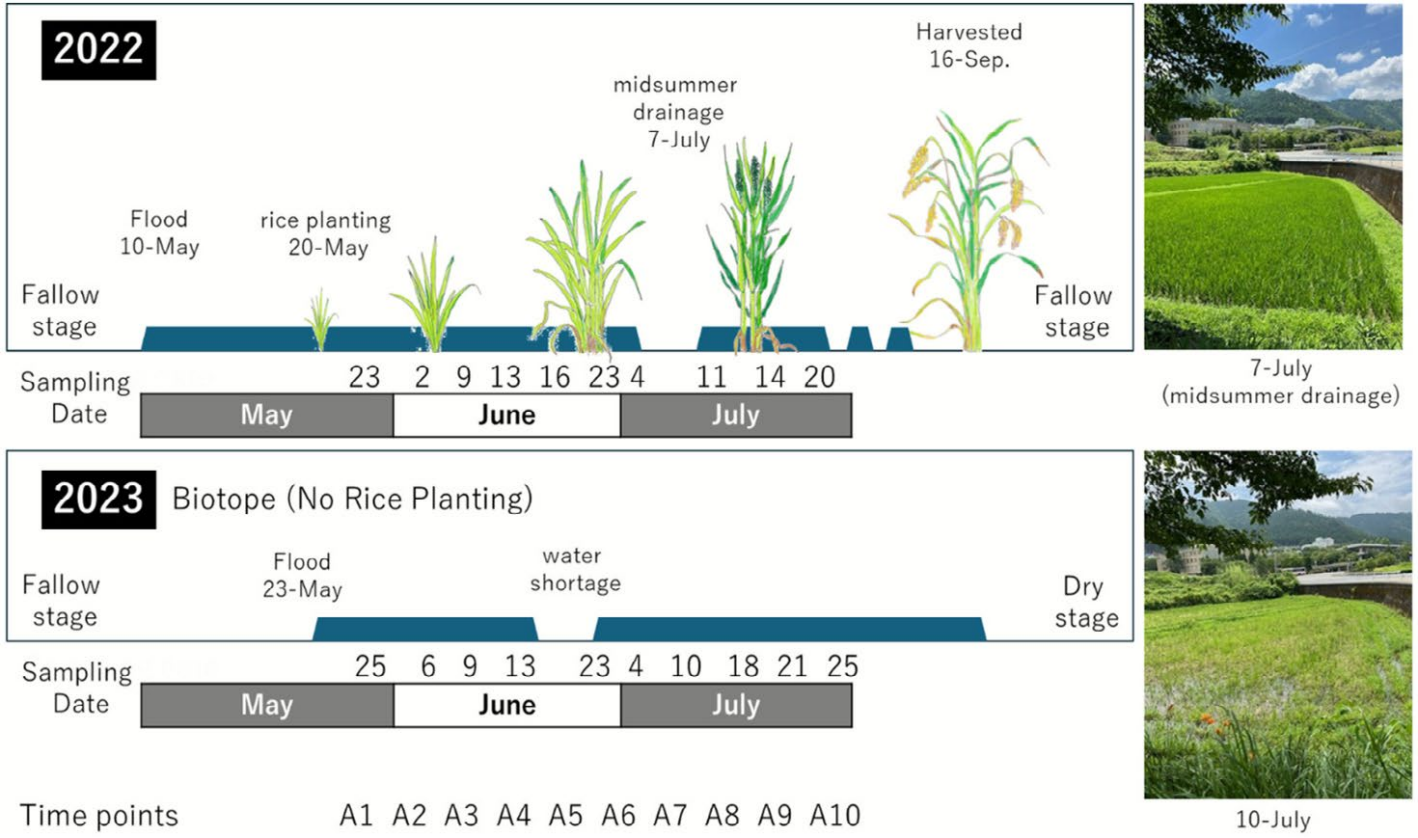
Comparison of study rice field between 2022 (cultivated) and 2023 (uncultivated, biotope condition). The blue trapezoid indicates water coverage in the field. The survey dates are designated sequentially as time points A1 to A10 for use in the figures and tables.

The study field was located in Kameoka City, Kyoto Prefecture, Japan (34° 99'N, 135° 55'E). The surveyed rice field is located in a basin within a paddy field area situated at the foot of the mountains on the outskirts of Kameoka City. The single rice field (0.07 ha) had been cultivated a long time before being abandoned in 2023. In 2023, rice cultivation was not carried out in this rice field, but as in previous years, irrigation resulted in a wetland‐like biotope that was flooded with river water (Figure 1). Plankton sampling was conducted between late May and July. The climatic conditions surrounding the surveyed rice field are characterized by a cool temperature in May, precipitation in June, and a gradual increase in temperature in July as the summer season commences. These periods correspond to the time the rice paddies are filled with water. After the survey period, the rice field was either not filled with water or had unstable irrigation; therefore, plankton surveys were not conducted. Generally, Japanese rice paddies are completely drained in winter, and water is drawn from rivers when it is time to plant rice. The only human‐induced alterations before and after abandonment were the non‐plowing of the soil and the non‐application of fertilizers or pesticides. Sampling frequency was conducted approximately once a week, for a total of ten times each year (2022 and 2023). While sampling every other day is recommended to evaluate how the plankton community responds to disturbances (Edson and Jones 1988; Padisák et al. 1988), the surveys were conducted at the same time each year to capture annual variations in this study.

In general, rice paddies in Japan are completely dried out in winter, and water is drawn in from rivers when it is time to plant the rice. In Japan, there is a traditional rice cultivation method in which rice paddies are completely drained and dried to make the root of rice plants strong. Normally, this drying period lasts for 7 to 10 days, but it varies depending on the precipitation and the farmers in each region. The rice paddy surveyed had been cultivated using this traditional method for decades, but in this year, there was heavy rain of over 30 mm around the time of the mid‐season drying; therefore, the period when the soil was completely dried out was short (3 days, from 5th to 7th July in 2022). On the other hand, in 2023, after the river water was supplied in late May, rice was not planted, and the field became a biotope. The water remained in the biotope at all times except for one instance of water shortage on 10th May, with weeds growing well the entire time. When collecting the water samples from the rice field, a total of five environmental variables were measured—water temperature (thermistor sensor, CUSTOM Corp., Tokyo), temperature (environment meter, AHLT‐102SD, CUSTOM Corp., Tokyo), pH (LAQUAtwin, HORIBA Ltd., Kyoto), and water depth (leveling rod, Shinwa Rules Co. Ltd., Niigata).

### 2.2 Plankton Sampling and Identification

Water was collected while walking along the shore of the rice field using a disposable cup (200 mL, polypropylene) over 20 points. Water samples were taken at multiple points due to the heterogeneous distribution of plankton caused by inflow and outlet ports, as well as wind (Fujita and Nakahara 1999; Yamazaki et al. 2004). At each sampling time, approximately 3 L of water was placed in a zippered bag (LZ, Seisannipponsha Ltd., Tokyo), and 1 L was filtered through a plankton net (NXX 25, Rigo, Tokyo). This 1 L of filtered water was used as a quantitative sample. After this sample (about 100 mL) was preserved in 1% Lugol's solution, it was used for quantitative analysis of phytoplankton and zooplankton. The remaining water was filtered using a plankton net and fixed in the same manner as the sample, and this was used as a subsample. The plankton were observed under a microscope (CX23, Olympus Corp., Tokyo) for all samples (20 samples) during periods of stable waterlogging (from May 23 to July 20, 2022; from May 25 to July 25, 2023).

The planktons targeted were phytoplankton: SAR (Alveolata, Rhizaria, and Stramenopiles), Bacteria (this study applies only to cyanobacteria), Euglenozoa, Chlorophyta, and zooplankton: Amoebozoa, Rotifera, Branchiopoda, and Copepoda (classification based on Urry et al. 2021). Identification by plankton morphology was based on a Japanese illustrated guide to morphological classification of zooplankton and phytoplankton (Tanaka 2022), to species as far as possible. For the morphology of protists that live in Japan, we referred to a pictorial book (Tsukii 2010) and its online database (Protozoa Information Server 2018, http://protist.i.hosei.ac.jp/taxonomy/menu.html). In particular, Copepoda were separated by growth stage (nauplius or adult), but were not identified to species level as dissection was required. Filamentous cyanobacteria were classified as cyanobacteria as it was not possible to classify them to the genus level. The abundance (ind l^−1^) of the plankton communities (including cells, individuals, and filaments) was measured three times for each sample using a 1‐mL plankton counting chamber (Matsunami Glass Ind. Ltd., Osaka, Japan).

### 2.3 Data Analysis

For the year‐to‐year comparison of plankton abundance and number of species/taxa, a t‐test was performed using R (R Core Team 2022). All other analyses were conducted using R version 4.3.3 with the “vegan” and “*ggplot2*” packages (R Core Team 2022). As with many studies in the field of ecology, this research adopted a significance level of *α* = 0.05 (Welch's t‐test). This is based on the standard practice of limiting the probability of rejecting a null hypothesis that is actually true to 5% or less. Community similarity across sampling dates was visualized using non‐metric multidimensional scaling (nMDS) based on Bray–Curtis dissimilarity. The Shannon‐Wiener index (*H′*) of the plankton community was calculated each year based on the count data. There are seven environmental variables: temperature (Temp), water temperature (WT), pH, water depth (Depth), the length of the hydroperiod, number of flooded days (Flooding), before or after it dried up, and conventional or biotope. To test whether there were statistically significant differences in plankton community structure (species composition) between the two years, we employed permutational multivariate analysis of variance (PERMANOVA), a multivariate analysis of variance. The analysis utilized species composition data (count data) from each sample. Dissimilarity (distance) between samples was calculated using the Bray–Curtis dissimilarity, commonly used for ecological data. The model was set as a one‐way design with year (2022, 2023) as a fixed factor. The analysis was performed using the “adonis2” and “simper” functions from the “vegan” package (version 2.8–0, https://vegandevs.github.io/vegan). The significance level was set at *α* = 0.05. Using the simper function to analyze the causes of any significant differences detected by PERMANOVA between communities in detail, we identified which species contributed most significantly through the “simper” function.

## 3 Results

### 3.1 Environmental Factors of Rice Field

**FIGURE 2:**
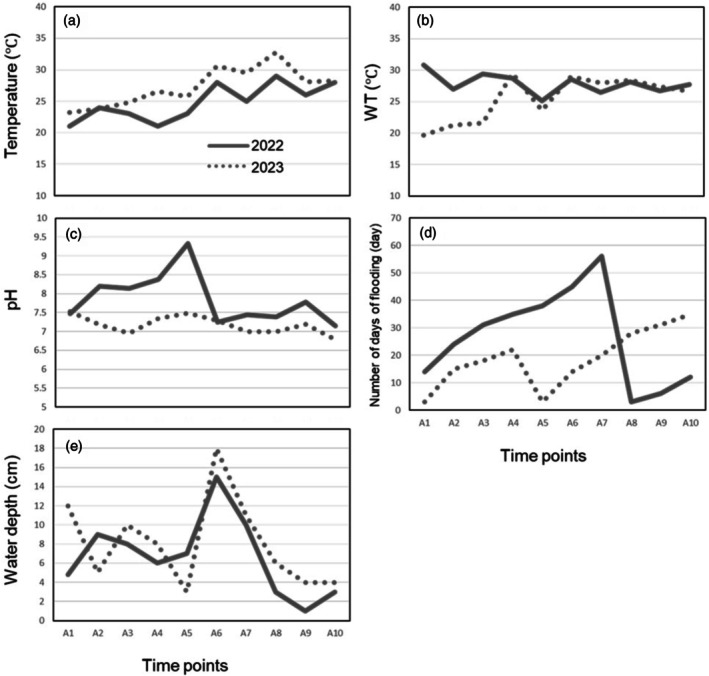
Environmental variables for each sampling date: (a) temperature, (b) water temperature, (c) pH, (d) number of flooding days, and (e) water depth. The horizontal axis of each graph is the sampling dates. The sequence A1 to A10 corresponds to the following dates: May 23, June 2, 9, 13, 16, 23, July 4, 11, 14, 20, 2022; and May 25, June 6, 9, 13, 23, July 4, 10,18,21,25, 2023.

The operational calendar and sampling dates for the paddy fields are listed in Figure 1. Figure 2 shows the environmental variables when the rice field was converted into a biotope. The water temperature profiles of the rice fields were almost the same in both years, with a few exceptions, although the outside temperature was higher in 2023 (Figure 2A). The pH was significantly higher in 2022 and peaked on 16 June, but was constant in 2023 (Figure 2C). The length of the hydroperiod in 2022 and 2023 was a maximum of 56 days and 35 days, respectively (Figure 2D). Water levels fluctuated due to artificial manipulation in 2022 and weather conditions in 2023 (Figure 2E).

### 3.2 Plankton Community During the Flood Rice Field Season

Over the two‐year periods of this study, we identified a total of 243 taxa, 200 of which were identified at the species level. Among these, three belonged to the phylum Alveolata, 80 to the phylum Stramenopiles, and two to the phylum Rhizaria, four to the Bacteria (cyanobacteria), 35 to the phylum Euglenozoa, 67 to the phylum Chlorophyta, 17 to the phylum Amoebozoa, 24 to the phylum Rotifera, ten to the class Branchiopoda and one to the subclass Copepoda (Table S1). In 2022, the maximum and minimum numbers of species/taxa were 45 (23rd June) and 14 (2nd June; 20th July), respectively, and in 2023, they were 72 (25th July) and 14 (25th May), respectively. The average number of species/taxa increased from 24.8 taxa in 2022 to 38.0 taxa in 2023. However, there was no statistically significant difference between the two years (*t*‐test: *t* = −1.86, *p* = 0.07).

**FIGURE 3:**
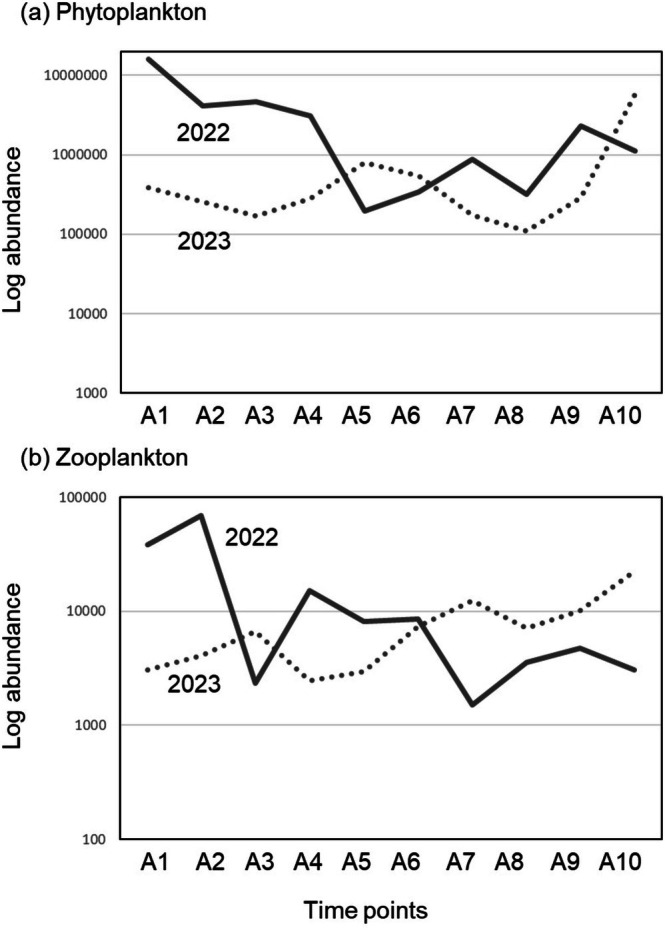
Information on sampling days (time points: A1–A10) for 2022 and 2023: Flooding days, and plankton diversity (bar chart, Shannon‐Wiener index, *H′*).

The biodiversity index (Shannon‐Weiner index, *H*′, Figure 3) for each year was calculated using count data. The highest diversity index was observed in 4.73 (22A8) on 11th July, 2022, and 5.10 (23A5) on 23rd June, 2023, both of which were immediately after experiencing drought. The average values for the diversity index in 2022 and 2023 were 2.41 and 2.78, respectively, and were slightly higher in the years when the land was left fallow.

**FIGURE 4:**
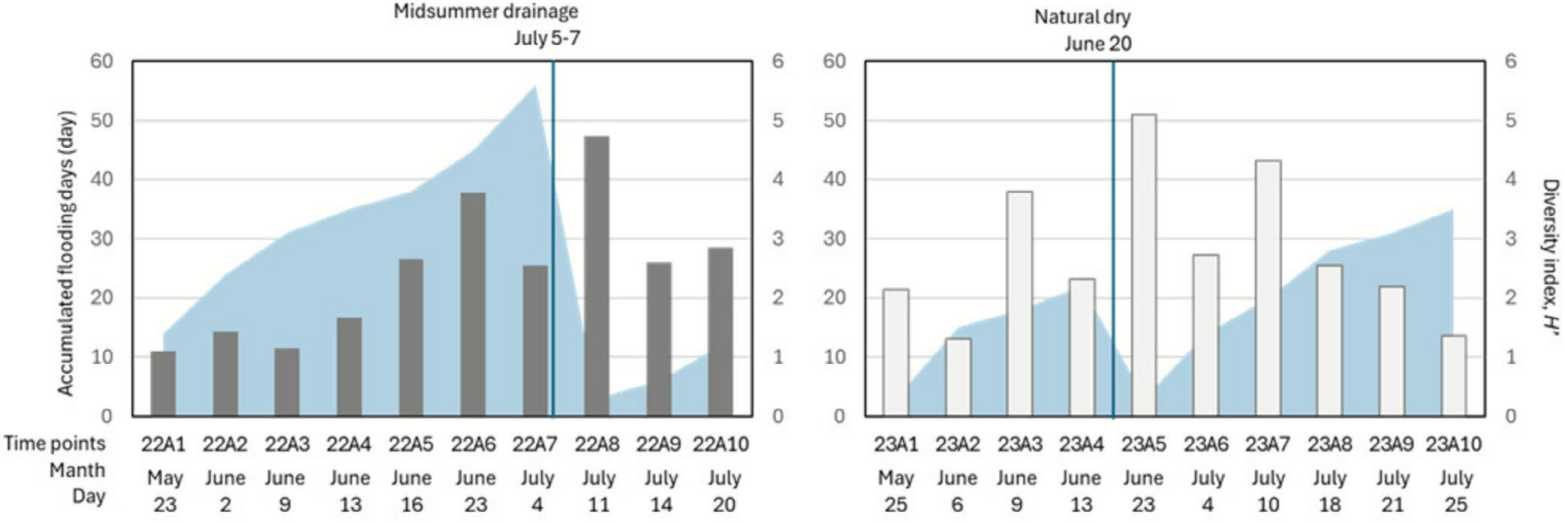
Changes in plankton log‐transformed abundance during the flooding period in 2022 and 2023. (a) Phytoplankton (cells l^−1^) (b) Zooplankton (individuals l^−1^). Solid lines represent 2022; dashed lines represent 2023.

The abundance of phytoplankton and zooplankton tended to be higher in 2022 than in 2023 (Figure 4). However, this was not statistically significant (*t*‐test, phytoplankton, *t* = 1.52, *p* = 0.116; zooplankton, *t* = 1.06, *p* = 0.30). The seasonal changes in phytoplankton and zooplankton were similar, with a downward trend in the latter half of the season in 2022 and an upward trend in the latter half of the season in 2023. The average abundance of each plankton is as follows: Phytoplankton (cells l^−1^): 3.0 × 10^6^ and 8.6 × 10^5^; zooplankton (ind. l^−1^): 4.1 × 10^4^ and 2.1 × 10^4^ in 2022 and 2023, respectively.

**FIGURE 5:**
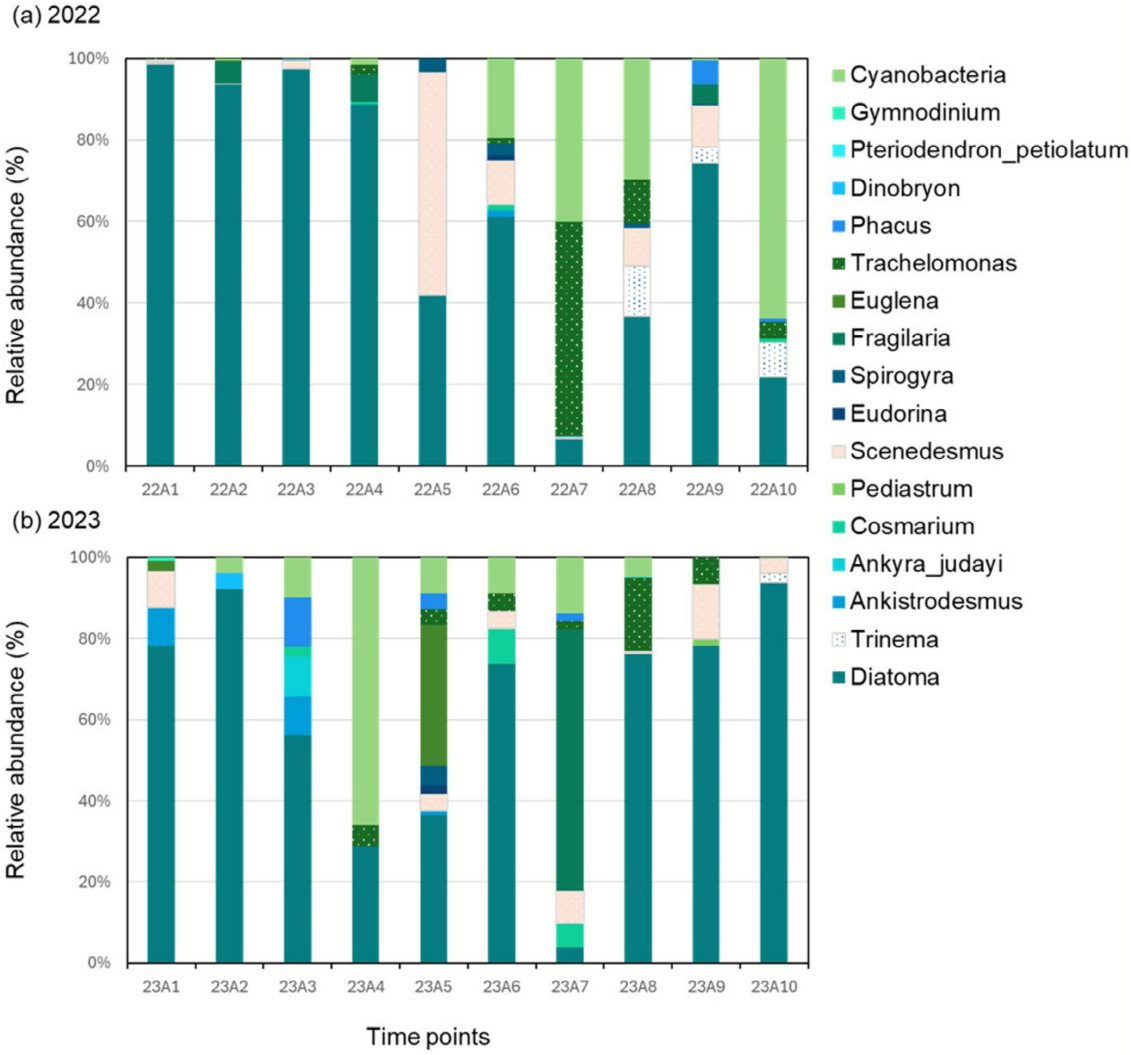
The relative abundance of phytoplankton taxa in (a) 2022 (rice paddy) and (b) 2023 (biotope).

**FIGURE 6:**
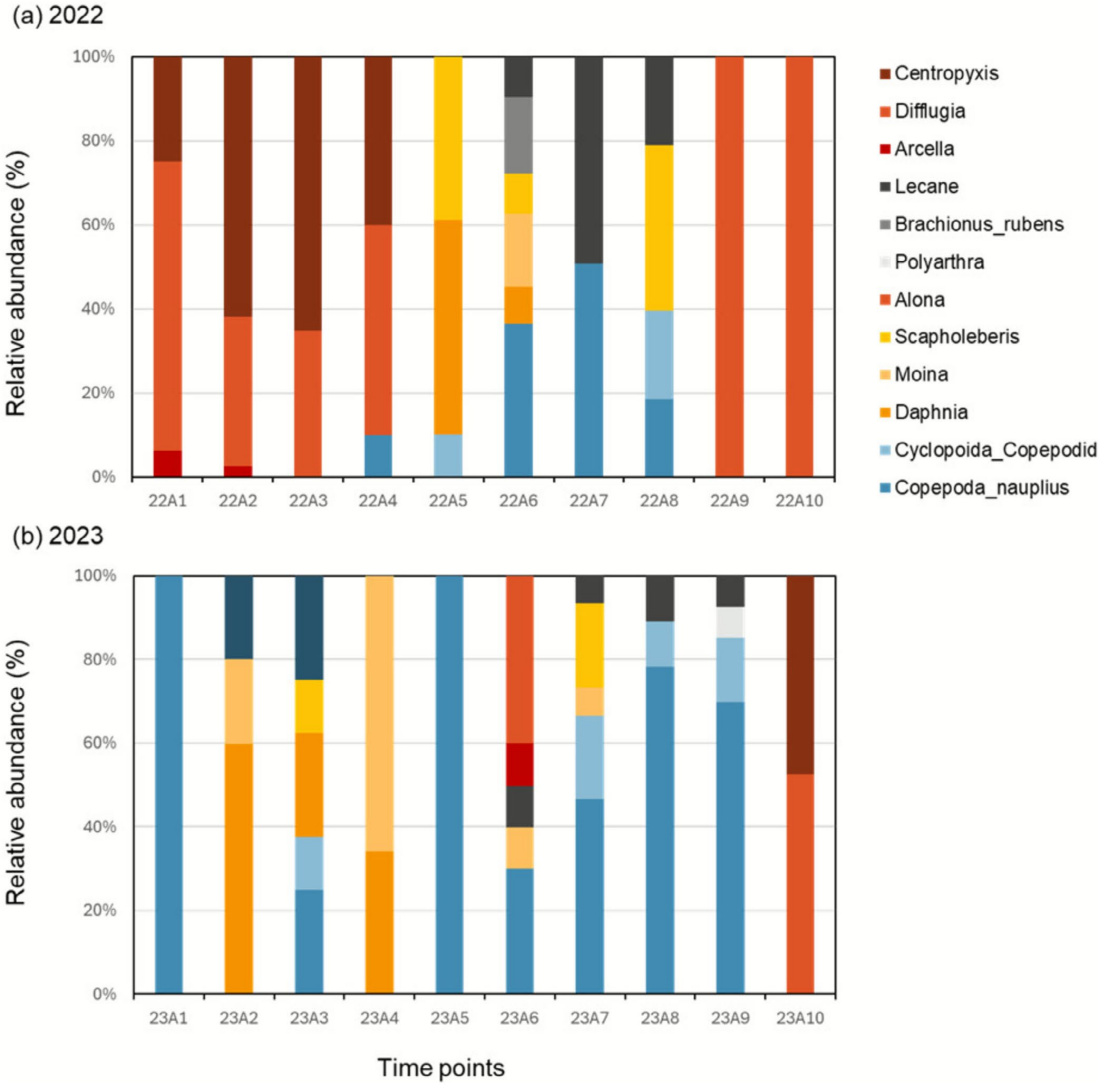
The relative abundance of zooplankton taxa in (a) 2022 (rice paddy) and (b) 2023 (biotope).

All plankton samples were composed of phytoplankton and zooplankton (Figures 5 and 6). *Diatoma* sp. was observed to be the dominant species in the field across both the years and almost all the sampling dates (Figure 5). In contrast, seasonal changes in zooplankton varied from 2022 to 2023, with the dominant taxa changing from testate amoeba (*Centropyxis*, *Difflugia*, and *Arcella*) to Arthropoda, Cyclopoida (Figure 6). The ratio of Rotifera was low in both years.

**FIGURE 7:**
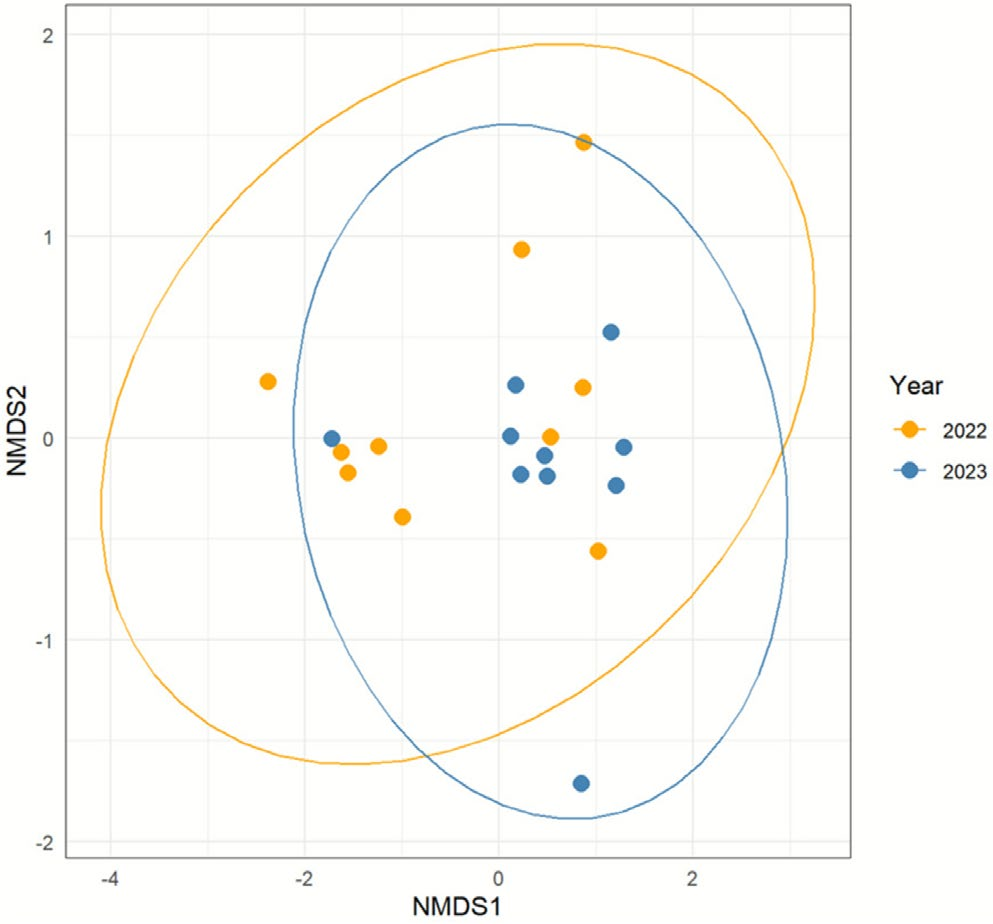
Non‐metric multidimensional scaling plot (nMDS) plot comparing plankton community composition between 2022 (yellow) and 2023 (blue) (stress = 0.082, permutation = 999; PERMANOVA R2 = 0.99, *p* = 0.097).

From the results of the count of plankton data, Bray–Curtis similarity (nMDS) was performed and illustrated for community similarity across the sampling dates (Figure 7). The stress values for the nMDS were 0.082, which were sufficiently low, suggesting that the data could be projected well in a two‐dimensional space. No significant differences in population composition were observed between 2022 and 2023 (PERMANOVA *R* ^2^ = 0.99, *p* = 0.097). In other words, the differences observed over the two years are likely within the range of random variation. The correlations between the nMDS plots of the plankton community and temperature were marginally significant (*r* = 0.24, *p* = 0.099). There was also a slightly significant correlation between community composition and cultivation methods (*r* = 0.13, *p* = 0.081, conventional in 2022 and biotope in 2023). However, the correlations with the other environmental variables were not significant (Temperature *p* = 0.09; Water temperature *p* = 0.41; pH *p* = 0.61; Water depth *p* = 0.54). In addition, in both years, there was disturbance in which the water drained out artificially or naturally and then re‐entered, but it was found that this did not affect the community (*r* = 0.01, *p* = 0.69). Statistical results showing no detectable differences between populations indicate that no plankton can characterize the population among the years. Subsequent SIMPER analysis revealed species with lower *p*‐values when grouped by year compared to when not grouped (*Trinema p* = 0.008; *Trachelomonas p* = 0.012; *Scenedesmus p* = 0.045, Table 1). This indicates that these species contribute statistically to the differences between the two years. *Diatoma* showed high contribution rates (64%) to the community as a dominant species.

**TABLE 1:** Plankton ranked abundance SIMPER contributors to % dissimilarity in plankton composition the average overall dissimilarity among sampling days (A) and among years (B).

(A) Non‐grouped contributions		(B) Grouped by year contributions		
Taxon	Relative contributio*n* (%)	Taxon	Relative contributio*n* (%)	*p*
Diatoma	0.638	Diatoma	0.637	0.163
Cyanobacteria	0.123	Cyanobacteria	0.131	0.103
Trachelomonas	0.055	Trachelomonas	0.062	0.012*
Scenedesmus	0.047	Scenedesmus	0.051	0.045*
Fragilaria	0.036	Fragilaria	0.03	0.924
Euglena	0.026	Trinema	0.022	0.008**
Trinema	0.018	Euglena	0.019	0.988
Phacus	0.013	Phacus	0.011	0.944
Cosmarium	0.009	Cosmarium	0.007	0.995
Ankistrodesmus	0.008	Spirogyra	0.007	0.123
Spirogyra	0.007	Ankistrodesmus	0.006	0.988
Ankyra_judayi	0.003	Copepoda_nauplius	0.003	0.910
Copepoda_nauplius	0.003	Ankyra_judayi	0.002	0.979
Sum	0.986	Sum	0.988	

**p < 0.001, *p < 0.05.

## 4 Discussion

This study clarified how the diversity of plankton communities changed pre‐ and post‐abandonment of a single rice field. No statistically significant changes in plankton communities were observed over the two years (Figure 7). This may be because there were no changes in the environmental parameters measured during this period, albeit few in number (Figure 2). Alternatively, the limited number of surveys (10 per year) may have resulted in insufficient data to detect true differences. It could be argued that it failed to capture even annual fluctuations. The only human‐induced changes before and after abandonment were that the soil was not plowed, and no fertilizers or pesticides were applied. No maintenance work, such as mid‐season drainage, was carried out either. There was a slight increase in the number and diversity of plankton species/taxa in the biotope, though it was not statistically significant. The lack of impact on plankton diversity and abundance even after the land‐use change to a biotope is thought to be due to the persistence of previous conditions. Alternatively, plankton may possess high resilience to this fluctuation or exhibit characteristics that make them resistant to such changes. Evaluating whether biotope creation contributes to diversity requires surveys of diverse paddy field samples, sufficient sample numbers before and after conversion, and repeated experiments. However, it is uncommon to have the opportunity to study diversity during periods of land‐use change. This study is one of the few reports that has elucidated this rare phenomenon through analysis of plankton communities.

Plankton diversity remained unchanged even after the creation of the biotope (Figure 7). However, the most significant finding in this study was the three taxa (*Trinema*, *Trachelomonas*, and *Scenedesmus*, Table 1) that showed significant differences when grouped before and after the biotope creation. Multivariate analysis revealed important qualitative changes that could not be distinguished using a single diversity index. These may simply be annual variations, but it was concluded that these taxa did indeed change between these two years. Since environmental factors remained largely unchanged from 2022 to 2023, the environmental changes experienced by the plankton community may have been gradual. The changes in the phytoplankton community that we observed in the fallow fields may have captured a regime shift. Phytoplankton are among the most sensitive aquatic organisms to environmental changes, often responding more rapidly and strongly than other planktonic groups. When a specific threshold is exceeded, the ecosystem may suddenly shift to another stable state, a phenomenon known as a regime shift (Blöcker et al. 2023). Regime shifts can cause a loss of biodiversity and a decline in ecosystem services, and they can have a significant impact on the socio‐economy. Previous studies have reported several cases of regime shifts in lakes (Mittelbach et al. 2006) and marine ecosystems (Daskalov et al. 2007), and it has been suggested that climate change and human activity are the main causes. However, the mechanisms of regime shifts are not fully understood, and the prediction and management of regime shifts in land‐use change is an urgent issue. If changes in this plankton community affect higher trophic levels immediately, such as aquatic insects, insect larvae, and amphibian larvae, the rice paddy landscape may change.

In order to ascertain whether the alterations in plankton community structure evident in this study signify a regime shift, it is imperative to undertake prolonged investigations. In addition to changes in biotope formation, this study included an artificial disturbance called “midseason drainage,” and the dynamics of plankton before and after this disturbance were noteworthy. In conventional farming in 2022, the greatest biodiversity was observed after the mid‐season drying (July 11, 2022), and a similar trend was observed after drying up in the biotope (June 23, 2023) (Figure 3). Similar trends have been reported in plankton diversity in rice fields in other regions (Yamazaki et al. 2003). The disturbance caused by drainage was not lethal to the plankton community, regardless of its cause, whether human or natural. This phenomenon may be attributed to its prolonged adaptation to the paddy field environment. Therefore, in the process of converting fallow fields into biotopes, it is possible that plankton will adapt to water withdrawal. This adaptation could potentially eliminate the need for year‐round flooding. A comprehensive investigation is necessary to determine whether biotope formation disrupts the plankton community or contributes to its stabilization, as in the original wetland. Implementing biotope creation requires, specifically, conducting surveys targeting diverse paddy fields in various regions, sufficient sample numbers before and after transition, and repeated experiments.

By converting abandoned land into biotopes, as demonstrated in this study, it is possible to maintain plankton populations. Given the existence of both highly diverse and low‐diversity paddy fields, this effect should be verified across various regions. For example, comparing rotifer species richness in Thai paddy fields, the study site had significantly fewer species, only about half (Thailand: 50 species according to Roger et al. 1991; this study: 24 species). Reports within Japan confirm 127 rotifer species (Database of Biota in Rice Paddies and Adjacent Areas 2020), none of which are considered rice paddy endemic (Hayashi 2016). The emergence of diverse plankton species may indicate high local diversity. The three species identified in this study, *Trinema*, *Trachelomonas*, and *Scenedesmus*, while contributing little to the community, can be interpreted as rapid‐response species. Testate amoebae like *Trinema*, in particular, are known to function as early warning signals for environmental disturbances in peatland (Marcisz et al. 2016). This response by testate amoebae was also observed in paddy fields, suggesting it is likely a common phenomenon.

## 5 Conclusion

Agricultural land abandonment and land development are global issues; however, their impacts on biodiversity remain unclear. This study is the first to clarify how the abandonment of rice fields, at the very moment it occurred, affected plankton communities. Specifically, after the rice field was abandoned and converted into biotopes, the plankton diversity index increased slightly, but this was not statistically significant. Comparing changes in the communities during active paddy field use and after biotope conversion revealed that three species (*Trinema*, *Trachelomonas*, and *Scenedesmus*) responded, unlike when analyzed separately. As this study is based solely on two years of investigation in a single paddy field, the universality of this phenomenon warrants further examination. Biotope conversion of abandoned paddy fields, though, does not show a clear reduction in diversity. Therefore, managing biotopes while focusing on species richness and testate amoebae like *Trinema* may be effective for biodiversity.

## Author Contributions

**Mariko Nagano:** conceptualization (lead), data curation (lead), formal analysis (lead), funding acquisition (lead), investigation (lead), methodology (lead), supervision (lead), validation (lead), writing – original draft (lead), writing – review and editing (lead). **Ryoma Teramoto:** investigation (supporting), writing – original draft (supporting), writing – review and editing (supporting).

## Funding

This work was supported by the Japan Health Foundation.

## Conflicts of Interest

The authors declare no conflicts of interest.

## Supporting information


**Table S1:** List of plankton in a single rice field in Kyoto over two years (rice planted in 2022, post‐biotope in 2023).

## Data Availability

The data supporting the findings of this study can be freely accessed at figshare: https://figshare.com/s/46befee24607e632e179.
