# Correction to “Impact on Plankton Communities Following Abandonment of Rice Cultivation and Biotope Creation”

**DOI:** 10.1002/ece3.73198

**Published:** 2026-03-01

Nagano, M., and R. Teramoto. 2025. “Impact on Plankton Communities Following Abandonment of Rice Cultivation and Biotope Creation.” *Ecology and Evolution* 15, no. 12: e72575. https://doi.org/10.1002/ece3.72575.

In paragraph 5 of the “Results” 3.2 section, the statement “No significant differences in population composition were observed between 2022 and 2023 (PERMANOVA *R* ^2^ = 0.99, *p* = 0.097).” contained an error in the numerical values. The correct *R* ^2^ value should be “*R* ^2^ = 0.099.” Similarly, the *R* ^2^ value in the caption for Figure 7 should also be “*R* ^2^ = 0.099.”

We apologize for this error.

